# Genome-wide interplay between chromatin and the transcription machinery

**DOI:** 10.1186/1756-8935-6-S1-O19

**Published:** 2013-03-18

**Authors:** B Franklin Pugh

**Affiliations:** 1 Penn State University, USA

## 

With the advent of high throughput and high resolution genome-wide protein-DNA detection assays, the inter-relationships between chromatin and the transcription machinery are now becoming clearer. Here I will discuss our recent findings using MNase ChIP-seq to map nucleosome positions, and an ultra-high resolution mapping technique called ChIP-exo that we recently developed (Rhee and Pugh, Cell 2011 14:1408). What is apparent from these studies is the following: 1) transcription factors bind to many more locations in the genome than previously appreciated. 2) transcription complexes form at the interface between nucleosomes and nucleosome-free promoter regions. 3) Chromatin remodeling complexes target specific nucleosome positions, working in concert to organize nucleosomes at the beginning and end of genes. Many remodeler subunits interact asymmetrically with the nucleosome core across the genome, which may be important for the directional passage of RNA polymerase II.

